# Executive functioning and divergent thinking predict creative problem-solving in young adults and elderlies

**DOI:** 10.1007/s00426-022-01678-8

**Published:** 2022-04-02

**Authors:** Alice Cancer, Paola Iannello, Carola Salvi, Alessandro Antonietti

**Affiliations:** 1 Department of Psychology, Catholic University of the Sacred Heart, Milan, Italy; 2 Department of Psychiatry and Behavioral Sciences, University of Texas at Austin, Austin, TX USA

## Abstract

The role of executive functioning in creative thinking is under debate. Some authors suggested that increased inhibitory control, a component of executive functioning, is detrimental to creative solutions, whereas others argued that executive functions are central to creative problem-solving, thus questioning Guilford’s classical distinction between divergent and convergent thinking. Executive functions decline with age. In this study, we investigated the contributions of executive functioning and its age-related decline and divergent thinking to creative problem-solving. To this aim, we divided our sample of sixty healthy adults into two age groups of young adults (20–26 years) and elderly (60–70 years) and we assessed their creative problem-solving abilities (using the compound remote associate problems) as well as other potential cognitive predictors of creative problem-solving (i.e., impulsivity, divergent thinking, verbal working memory, and decision-making style). A linear regression model revealed that the ability to solve problems creatively is negatively predicted by older age and impulsivity, while positively predicted by divergent thinking and verbal working memory. These findings reveal a combined contribution of executive functions and divergent thinking to creative problem-solving, suggesting that both convergent and divergent processes should be considered in interventions to contrast age-related decline.

## Introduction

Creativity is a multifaceted construct, defined as the ability to generate novel ideas that are not only original and unusual, but also relevant, appropriate, and useful (Runco & Jaeger, 2012). Such a combination of originality and effectiveness provides both individual and societal benefits (Hennessey & Amabile, 2010) and supports advancements in various disciplines. Understanding the neuropsychological basis of the creative process is particularly relevant to facilitate the identification and development of creative thinkers and problem-solvers.

The literature presents mixed results about the role of executive functioning in the creative process (Hennessey & Amabile, 2010). Some researchers suggested that “superior executive functioning, such as increased attentional control, may be detrimental to reaching creative solutions” (e.g., Jarosz et al., 2012), thus implying that relatively less executive engagement would overall enhance creativity (Wiley & Jarosz, 2012). Problems can be solved creatively or via step-by-step analysis. Creative problem-solving is based on solution processes that are divergent, associational, and discontinuous (Runco, 2014) and it is obstructed by inhibition (Radel et al., 2015). Problem-solving via analysis instead requires the use of algorithms and step-by-step above awareness procedures that are facilitated by inhibition, shifting, and working memory (WM) capacity (Kane et al., 2004; Viterbori et al., 2017; Zelazo et al., 1997).

Creative problem-solving, which involves connecting weakly related and remote concepts, is facilitated by producing many alternative responses, re-organizing the problem space by de-contextualizing its elements, and connecting the ideas through unusual combinations (Antonietti & Colombo, 2013). According to the hypothesis of a detrimental role of executive functioning in creative problem-solving, increased attentional control would negatively interfere with such processes. A state of diffused attention facilitates internal focus, as well as the retrieval of weakly activated and irrelevant concepts, and provides original solutions to problems (Ansburg & Hill, 2003; Carson et al., 2003; Dykes & McGhie, 1976; Salvi & Bowden, 2016; Salvi et al., 2015). Several works based on neurophysiological markers are showing that creative problem-solving is associated with a state of disengagement from the external inputs (Jung-Beeman et al., 2004; Kounios et al., 2006, 2008; Salvi et al., 2015, 2020). When people are engaged in thinking creatively, they tend to gather distracting information by closing their eyes or by looking toward an empty space or a blank wall. This “looking at nothing” behavior is commonly understood to be a way to avoid distracting information so that one can concentrate on inner thoughts (Salvi & Bowden, 2016). Results from Jarosz et al.’s study (2012) supported this negative association, showing a positive effect of alcohol intoxication on RAT accuracy and speed, through lower attentional control and lower working memory (WM). Findings from clinical studies on creativity pointed to similar conclusions (Abraham, 2019). Patients with focal damage to the frontal cortex, a brain area that is typically involved in executive functions (Duncan, 2001), were shown to outperform healthy control in an insight problem-solving task (i.e., the matchstick arithmetic task, Knoblich et al., 1999) (Reverberi et al., 2005). Furthermore, high levels of creativity have been reported in Tourette’s patients, which were explained as the result of altered connectivity patterns between the frontal and prefrontal cortex (Colautti et al., 2021).

There is, however, a growing body of research showing that executive functions are central to creative thinking (Nusbaum & Silvia, 2011). According to Nusbaum and Silvia (2011), divergent and convergent thinking are more closely related than the classical Guilford’s (1967) distinction presumed—namely, the expansive generation of novel ideas in contrast with the selection of a unique response from several possible alternatives (Guilford, 1967). Using latent variable modeling, Nusbaum and Silvia (2011) found that executive shifting predicted a successful performance in a divergent thinking task, namely, the alternative uses task (AUT) where respondents are asked to list as many ways of employing a common object as possible (Nusbaum & Silvia, 2011; Silvia et al., 2009). Sharma and Babu (2017) reported a significant relationship between measures of creativity (i.e., Torrance Test of Creative Thinking—TTCT; Torrance, 1990) and inhibitory control, as measured by the Stroop test. However, no association was found between creativity and WM. Similar results were obtained by Edl et al. (2014) in a sample of design students, who showed a significantly lower Stroop interference effect as compared to a control group and a strong correlation between inhibitory control and creative thinking, as measured by TTCT (Torrance, 1990). According to the authors, these findings showed that inhibition is required to suppress dominant but irrelevant response tendencies during a creative task. Clinical evidence on dementia (Fusi, Crepaldi, et al., 2021; Fusi, Lavolpe, et al., 2021) showed that although patients with frontotemporal dementia could produce a great number of ideas, they were not able to flexibly combine the information to produce original ideas, due to damages in the prefrontal cortex. In addition, other findings highlighted that metacognitive control during the performance of a creative task, in the form of predictions/retrospections tasks, facilitates the broadening of the mental field (Antonietti et al., 2021), thus suggesting a combined contribution of convergent and divergent processes. Neuroscientific evidence supports the involvement of executive functions in creative thinking, showing a cooperative interaction of the default network (associated with mind-wandering and self-generated thought) and the executive control network (associated with working memory, relational integration, and task-set switching) during creative cognition (Beaty et al., 2016, 2019).

Executive functioning is particularly susceptible to advancing age (Craik & Grady, 2002; MacPherson et al., 2002). Cross-sectional (Rhodes, 2004) and longitudinal studies (Sapkota et al., 2017) established a negative association between aging and executive functioning in healthy adults. Extensive literature highlights that the combination of limited resources, reduced efficiency, and increased interference between tasks may often lead to deterioration of cognitive performance with aging (Lacour et al., 2008; Wollesen et al., 2016). The distinction between crystallized and fluid intelligence is often used to frame this cognitive decline over the lifespan. Fluid abilities (e.g., executive functions, processing speed, WM, response inhibition) tend to gradually decline over the lifespan (Salthouse, 2012) when compared with crystallized abilities (e.g., vocabulary, general knowledge), which tend to remain stable or even improve through the last decades of life (Salthouse, 2012).

Processing speed, which refers both to the speed of cognitive task performance and the speed of motor responses, continues to deteriorate starting from the third decade of life on (Salthouse et al., 1995), thus resulting in a slowed processing that can negatively affect performance across a variety of cognitive domains. Slowed processing speed, as well as reduced capacity to ignore irrelevant information and poor use of strategies, is partly related to age-related decline in WM (Luszcz & Bryan, 1999). Aging is also negatively associated with response inhibition, which is the ability to inhibit an automatic response in favor of producing a novel one (Wecker et al., 2000). Impulsivity has been found to increase together with age-related cognitive decline (Sakurai et al., 2020), thus highlighting a high level of impatience and impulsivity among elderly people (Read & Read, 2004).

Besides executive functions and age-related cognitive changes, another component of creativity, namely, divergent thinking, can be hypothesized to have a predicting role in creative problem-solving. Divergent thinking, through the generation of numerous (i.e., fluency) and unconventional (i.e., originality) ideas that are shifted into various content categories (i.e., flexibility) (Guilford, 1956), elicits creative solutions to problems (Barbot et al., 2019). The generation of divergent options increases the chance to find among them the correct solution to the problem, ultimately facilitating its identification.

This study aims to further the understanding of the complex relationships between executive functioning, divergent thinking, and creative problem-solving in adults and older adults. More precisely, we aimed to measure the role of inhibition, divergent thinking skills, WM, and age-related cognitive decline as predictors of creative problem-solving. We hypothesized that if executive functions have a role in creative problem-solving and divergent thinking, we would find a correspondent decline in elderly people compared to young adults.

## Materials and methods

### Participants

Sixty healthy participants volunteered to be involved in the study. The sample included two age groups: a subgroup of elderlies, aged 60–70 (*N* = 30) and a subgroup of young adults, aged 20–26 (*N* = 30).

Older participants were recruited among members of an association for the social promotion of elderlies in Milan, Italy. Younger participants were recruited among students attending non-humanistic University courses (i.e., architecture, engineering, law, economics, agriculture, and dentistry) in several institutions in Milan, Italy. For both subgroups, a voluntary response sampling method was used: the study was advertised on campus and at the elderly association.

Normal cognitive functioning, as measured through the Mini-Mental State Examination (MMSE) (Folstein et al., 1975) (corrected total score ≥ 24), was defined as the inclusion criterion for the elderly participants. Exclusion criteria for the whole sample were (a) medication with either tricyclic antidepressant or selective serotonin reuptake inhibitor (SSRIs); (b) dementia or neurological disorders; (c) non-native Italian language—due to the verbal nature of the primary outcome measure, namely, a verbal problem-solving task.

Participants’ written informed consent was obtained prior to recruitment. The study was conducted following the ethical principles laid down in the Declaration of Helsinki (World Medical Association, 2001).

### Assessment measures

#### Creative problem-solving

The Italian adaptation of the Compound Remote Associate (CRA) problems by Salvi et al. (2016), originally developed by Bowden and Jung-Beeman (2003), was used to measure creative problem-solving abilities. The task, inspired by the Remote Associate Test by Mednick (1968), includes 80 problems in the form of triplets of words. Participants are asked to find a solution word that forms either a compound word or a common two-word phrase with each of the three problem words (e.g., the solution to the triplet CRAB PINE SAUCE, is *apple*—which forms the compounds *crab apple*, *pineapple*, and *apple sauce*). A computerized version of the CRA problems was used. Participants had 15 s to complete each problem; if no answer was given before the time-out, an omission error was recorded. A CRA performance criterion (*C*) index was computed by subtracting the number of commission errors (i.e., incorrect responses) from the number of correct responses and dividing by the number of total CRA problems (*n* = 80). If *C* = 0, the subject’s criterion was ‘neutral’, showing no inclination towards the generation of either correct or incorrect responses. If *C* > 0, the subject generated a higher number of correct responses, whereas if *C* < 0, the subject generated a higher number of incorrect responses. We used the remote associates as a matter of consistency with the literature that drove our hypothesis and since they are a classic measure of creative problem-solving (e.g., Mednick, 1968; Shen et al., 2016, 2018).

#### Impulsivity

Self-reported impulsivity was measured through the Barratt Impulsiveness Scale (BIS-11) (Italian version: Fossati et al., 2001; Patton et al., 1995). The scale, that measures the personality/behavioral construct of impulsiveness, includes 30 items describing common impulsive (e.g., ‘I change hobbies’) or non-impulsive (e.g., ‘I plan trips well ahead of time’) behaviors, to be evaluated on a 4-point Likert scale (1 = ‘Rarely/Never’ to 4 = ‘Almost Always/Always’). The scale’s total score was considered.

Furthermore, the Go/No-go paradigm, namely, a computerized motor response control task, was used to measure inhibitory control together with focused and sustained attention. Participants were required to respond to the presence of a target letter, during a sequential presentation of letters, by pressing the space bar on a computer keyboard. The task included 396 trials, in which each letter (either W or M) was individually presented on a computer screen for the duration of 150 ms, with an inter-stimulus interval of 750 ms. In the first condition (198 trials, W-Go) participants were asked to press the space bar in response to the target letter W and withhold their response to the non-target letter M. In the second reversed condition (198 trials, M-Go), they were asked to press the space bar in response to the target letter M and withhold their response to the non-target letter W. The ratio of targets to non-targets was 70:30 in both conditions. A composite measure of attention and inhibitory control was computed as the proportion of accurate responses, including the correct responses to the target letter (hits) and the correct rejections to the non-target letter.

#### Divergent thinking

Divergent thinking was assessed using AUT (Guilford, 1967). Participants were asked to list as many possible uses for common items, such as a brick, a newspaper, or a spoon. A total of six items were individually presented, for which participants had 3 min to generate as many responses as possible. A fluency score was computed as the average number of alternative uses generated by the participant for each item. Furthermore, two independent judges rated the originality of each response (i.e., the extent to which the provided use is deemed divergent from the intended uses of that object) on a scale from 1 (not at all) to 5 (very). An originality score was computed by averaging the ratings by the two judges. The average originality score of the first generated response for each item was considered as a measure of immediate divergent thinking. We adopted a subjective scoring system, using raters blind to order of the responses (Hass & Beaty, 2018; Hass et al., 2018).

#### Verbal working memory

Verbal WM was assessed through the Digit Span subtest from the Wechsler Adult Intelligence Scale-Fourth Edition (WAIS-IV) (Italian version: Orsini & Pezzuti, 2013; Wechsler, 2008). The subtest includes three tasks, in which participants are asked to repeat a series of digits forward (i.e., Digit Span Forward), backward (i.e., Digit Span Backward), and in ascending order (i.e., Digit Span Sequencing). A Digit Span scaled score was computed based on Italian normative data.

#### Decision-making style

To measure individuals’ habitual preference for intuition—a thinking style that can be related to cognitive divergence (Iannello et al., 2020)—versus deliberation—a thinking style which, because of the reliance on analytical processing, can be related to convergent thinking—when making a decision, the Preference for Intuition and Deliberation (PID) scale (Betsch, 2004; Pachur & Spaar, 2015; Italian adaptation: Raffaldi et al., 2012) was used. The questionnaire includes 18 items (e.g., ‘I like situations in which I have to rely on my intuition’) to be answered on a 5-point scale (1 = ‘I don’t agree’ to 5 = ‘I completely agree’). Two separate scores representing the tendency to ponder decisions intuitively (i.e., Intuition subscale) or deliberately (i.e., Deliberation subscale) are computed.

### Procedure

Participants underwent two 90-min lab testing sessions. Tasks’ order was counterbalanced. Before completing the experimental procedure, the MMSE (Folstein et al., 1975) was administered to older participants to screen for any severe cognitive impairment.

### Statistical methods

First, descriptive statistics of participants’ socio-demographic characteristics were computed. Second, age group (young adults vs. elderlies) differences were tested on years of education, decision-making style, and CRA performance parameters (i.e., response time, correct responses, commission errors, omissions, criterion). Given that group variances were unequal (Levene’s Test *p* < 0.001) for most measures, Welch’s t test was used. The p value was adjusted to correct for multiple testing within measures of the same construct (i.e., CRA parameters, Alpha = 0.05/4 = 0.012).

Finally, a linear regression model was tested to measure the contributions of all predictors (i.e., impulsivity, creativity, verbal WM, decision-making style) to the CRA criterion index, which was assumed as the most relevant score of creative problem-solving performance. A sample size of 60 was calculated to be enough to detect a medium effect size (*η*^2^ = 0.2) in a linear multiple regression model with six predictors, with a power of 0.80 and alpha set at 0.05. The assumption of no multicollinearity was confirmed in all multiple regression models (all VIF values ranged between 1.02 and 1.57). Predictors were entered into the model using the forward method and they were selected by comparing the models’ goodness of fit (*F* tests) and considering AIC values. Effect sizes have been reported as Cohen’s *d*.

## Results

### Participants’ characteristics

The sample was composed of a subgroup of elderlies, aged 60–70 (*N* = 30, *M*_age_ = 64.9, SD 4.28) and a subgroup of young adults, aged 20–26 (*N* = 30, *M*_age_ = 23.8, SD 2.11). Gender was equally distributed between age groups (young adults: 60% female, elderlies: 66.7% females, *χ*^2^ = 0.29, *p* = 0.59).

In our elderly sub-sample, 43.3% of participants were retired and 56.7% were employed. They underwent formal education for 6–21 years (*M* = 13.2; SD 4.65) and they were employed for 0–50 years (*M* = 32.2; SD 13.2). The years of formal education of the younger participants ranged from 13 to 20 (*M* = 17.2; SD 1.83).

### Age differences in decision-making style and CRA parameters

Table 1 reports differences between age groups (young adults vs. elderly). The two groups differ in years of education, with younger participants being significantly more educated (*t* = 4.46, *p* < 0.001, *d* = 1.15). Given that the education variance was vastly explained by the age group (*β* = − 1.00, CI [− 1.45, − 0.55], *p* < 0.001), this measure could not be included in the subsequent regression model among the other predictors.

**Table 1:** Age group differences in years of education, decision-making style (PID scale), and CRA parameters

	Young adults, *N* = 30		Elderlies, *N* = 30		Welch’s *t* test		
	*M*	SD	*M*	SD	*t*	*p*	*d*
Years of education	17.23	1.83	13.17	4.65	**4.46**	**< 0.001**	**1.15**
Decision-making style							
Deliberation	3.76	0.56	3.95	0.60	− 1.24	0.22	− 0.32
Intuition	3.85	0.56	3.76	0.54	0.63	0.53	0.16
CRA parameters							
RT (ms)	9048.93	1527.21	5115.20	2949.88	**6.49**	**< 0.001**	**1.68**
Omissions	0.38	0.13	0.30	0.17	2.23	0.03	0.58
Correct responses	0.43	0.12	0.40	0.12	1.08	0.28	0.28
Commission errors	0.19	0.09	0.31	0.17	− **3.43**	**< 0.001**	**0.89**
Criterion index	0.25	0.17	0.09	0.24	**2.85**	**0.006**	**0.74**

Alpha = 0.012. Significant differences are marked in bold

Decision-making style, as measured by the two subscales of the PID questionnaire, was comparable between groups (preference for deliberation: *t* = − 1.24, *p* = 0.22; preference for intuition: *t* = 0.63, *p* = 0.53).

As for the CRA problems, we compared each parameter (i.e., response time, correct responses, commission errors, omissions, criterion) between age groups to explore the specific performance profile of young adults and elderlies. CRA response times were overall rather long, with high variability (*M*_ms_ = 7082; SD = 3059). Furthermore, short response times were found to be strongly associated with a higher commission error rate (*r* = − 0.58; *p* < 0.001). The comparison between age groups showed that the elderlies tended to respond significantly faster than the younger participants (*t* = 6.49, *p* < 0.001, *d* = 1.68). The omission rate (*t* = 2.23, *p* = 0.03) and the number of correct responses did not differ between groups (*t* = 1.08, *p* = 0.28). However, the elderlies produced significantly more commission errors (*t* = 3.43, *p* < 0.001, *d* = 0.89). A high commission error rate points to difficulties in managing impulsivity. Furthermore, the CRA criterion index showed a significantly poorer performance of elderlies, as compared to young adults (*t* = 2.85, *p* = 0.006, *d* = 0.74).

### Predictors of CRA performance

CRA performance, measured as the CRA criterion index (adj*R*^2^ = 0.46, *F*_1,49_ = 7.50, *p* < 0.01), was negatively predicted by age (*β* = − 0.41, CI [− 1.63, − 0.11], ns), impulsivity (self-report scale: *β* = − 0.15, CI [− 0.01, 0.001], ns; Go/No-go task accuracy: *β* = 0.38, CI [0.13, 0.61], *p* < 0.01), with inhibitory control uniquely contributing to the prediction. Furthermore, CRA performance was positively predicted by divergent thinking (fluency: *β* = 0.14, CI [− 0.01, 0.03], ns; originality: *β* = 0.09, CI [− 0.05, 0.10], ns) and verbal WM (*β* = 0.29, CI [0.005, 0.03], *p* < 0.01), with the last one uniquely contributing to the prediction (Table 2).

**Table 2:** Estimated regression coefficients and associated *t* tests for the model predicting the CRA criterion index

	CRA criterion index			
	*β*	SE	*t*	*p*
Age group				
Elderly–Young	− 0.41	0.05	1.63	0.11
BIS-11	− 0.15	0.002	1.29	0.20
Go/No-Go task accuracy	0.38	0.12	3.17	**0.003**
AUT fluency	0.14	0.01	1.22	0.23
AUT originality of the 1st idea	0.09	0.04	0.75	0.45
Verbal WM	0.29	0.01	2.74	**0.009**

Bolded *p*-values indicate significant alpha (*p* ≤ 0.01)

## Discussion

The results of our study provide further evidence on the debated role of executive functions in creative problem-solving. Because executive functions decrease with aging, we compared the problem-solving performance, as well as WM, impulsivity, and divergent thinking of a group of older adults to a one of younger adults. Results showed that problem-solving is negatively predicted by aging and impulsivity, and positively predicted by divergent thinking and verbal WM.

The novel approach which was implemented in our study consisted in testing divergent thinking, using the classical AUT, as a predictor of creative problem-solving. Such a decision was based on the assumption that divergent thinking is a predictor of creative responses (Runco & Acar, 2012) rather than a measure of creative thinking. Based on these premises, a two-componential structure of the creative process leading to generate a unique solution to a problem, such as in the CRA tasks, was hypothesized. We suggest that the process leading to creative problem-solving comprises two sequential steps: (a) a divergent phase and (b) a convergent phase. In the first phase, a great number of disparate options are rapidly generated, regardless of their appropriateness. In the second phase, the generated options is narrowed down by inhibition control, through the suppression of dominant but irrelevant responses, to find the unique appropriate solution to the problem. These steps mirror the two main features of creative productions, namely, originality and effectiveness (Runco & Jaeger, 2012). While a decreased executive functioning would be beneficial in the first step of the creative process, the second convergent phase requires the involvement of WM and inhibition control. The results of our study confirmed this assumption, showing that both divergent thinking and executive functioning predict a better performance in creative problem-solving. Such a hypothesis is consistent with the dual pathway model postulated by Nijstad et al. (2010). The model assumes that there are two pathways to creative performance: (a) the flexibility pathway, which assumes a flexible switch between broad cognitive categories, as well as the use of remote associations; (b) the persistence pathway, which requires the systematic and focused exploration of possibilities, and the in-depth exploration of limited task-directed perspectives. According to the authors of the dual pathway model (Nijstad et al., 2010), during creative problem-solving, individuals switch from more flexible to more systematic processing modalities, thus highlighting the combined contribution of both pathways. Further Beaty et al. (2019), based on the interaction of functional connectivity between different networks during the creative performance, identified three processes that would predict an individual’s creative ability and which are in support of our results: (a) goal-directed memory retrieval, namely, the ability to strategically search episodic and semantic memory for task-relevant information; (b) prepotent-response inhibition, namely, the ability to suppress interference from salient and/or dominant responses; and (c) internally-focused attention, namely, the shielding of internal processes from external interference.

In regards to age-related cognitive decline, a recent systematic review by Fusi et al. (Fusi, Crepaldi, et al., 2021; Fusi, Lavolpe, et al., 2021) highlighted the nonlinear and multidimensional nature of the relationship between aging processes and divergent thinking performances. Mixed results can be found in the literature depending on figural vs. verbal divergent thinking tasks, and when specific indexes are considered (i.e., originality, fluency, and flexibility). The authors of the review also underlined the role of WM and processing speed, which explained the discrepancies between younger and older adults. Overall, creative performances of the elderlies, particularly in the verbal domain, are comparable to those of younger individuals when no time constraints are set during the task and the workload is not too high (Fusi, Crepaldi, et al., 2021; Fusi, Lavolpe, et al., 2021). In our study, CRA problems imposed a time constraint to complete each problem (i.e., 15 s). To manage the limited time available to generate a response, the older participants in our sample responded faster than the younger participants. We interpreted this result as a strategy to compensate for the age-related decreased processing speed, by rapidly generating a greater number of incorrect responses in a shorter time. However, based on the available data, we cannot predict what would have happened if no time constraint was imposed in the CRA task.

Interestingly, the number of omission errors did not differ between the younger and older subsamples. Older participants showed a significantly lower overall performance in the creative problem-solving task and an increased number of commission errors. The higher commission error rate in the elderlies revealed an impulsive tendency, typical of age-related cognitive decline (e.g., Morales-Vives & Vigil-Colet, 2012), that was detrimental for the performance in the creative problem-solving task used in our investigation. This result is also confirmed by the significant and independent negative contribution of inhibition control (i.e., Go/No-Go task). We hypothesized that impulsivity, both as a personality/behavioral trait (as measured by BIS-11) and a neuropsychological ability (as measured by Go/No-Go task), would facilitate the production of disparate ideas in the first divergent phase of the creative problem-solving process, but that it would interfere during the second convergent phase, in which WM and inhibition control help discriminate between appropriate and inappropriate solutions.

As for the association between WM and creative thinking, the insignificant role of WM found by Sharma and Babu (2017) was consistent with Jarosz et al.’s (2012) results on intoxicated participants. Nonetheless, the task used for measuring creative thinking by the former, namely, figural TTCT, does not require heavy demands on WM capacity (Roskos-Ewoldsen et al., 2008). On the contrary, our results evidenced a positive association between verbal WM and creative problem-solving. The role of WM is twofold: (a) To maintain the novel information activated, and thus easily accessible during the resolution of a task; (b) To discriminate between task-related relevant and irrelevant information (Unsworth & Engle, 2007). We argue that both processes are crucial for solving CRA problems, especially in the last convergent phase of problem-solving. Such a result is consistent with the study by Colombo et al. (2018) on the relationship between creativity and cognitive reserve, who found a positive correlation between the digit span and the creativity performance in healthy adults.

One of the limitations of the study is having adopted verbal tasks to measure divergent thinking, WM, and creative problem-solving. Given the prevalence of verbal tasks, we controlled for participants’ decision-making style to exclude any possible effect of individuals’ preference for a verbal code on the task performance—analytical people tend to prefer processing information that is primarily verbal (Betsch & Iannello, 2009; Epstein et al., 1996; Raffaldi et al., 2012). Nonetheless, as a future direction, the predictions we found should be re-tested using nonverbal measures, to exclude a domain-related effect of executive functioning on creative problem-solving.

Furthermore, the discrepancy in the two investigated subsamples (i.e., younger, and older adults) made it impossible to control for educational level in our model. Nonetheless, previous research excluded that education plays a significant role in divergent and creative thinking (Palmiero, 2015).

## Conclusions

Our study furthered the understanding of the contribution of executive functions and divergent thinking to creative problem-solving. According to the classical definition of creativity, creative products are both original and appropriate (Runco & Jaeger, 2012). The results of our study highlight a combined role of divergent thinking and executive functions in the solution of creative problems.

Specifically, divergent thinking would support the generation of numerous original ideas and unusual associations, crucial in the first divergent step of the creative process, whereas inhibition control and WM would support the appropriateness assessment of the generated options to ultimately identify the unique appropriate solution.

In conclusion, to facilitate creative problem-solving processes, not only divergent thinking but also executive processes should be trained and improved by interventions. Such an approach would be especially functional to target the detrimental role of age-related cognitive decline in problem-solving.

## Data Availability

The datasets generated during and analysed during the current study are available from the corresponding author on reasonable request.
